# Heart Failure in Poland: A 20-Year Epidemiological Perspective

**DOI:** 10.3390/medicina61081472

**Published:** 2025-08-16

**Authors:** Michał Bohdan, Anna Kowalczys, Jadwiga Nessler, Ewa Straburzyńska-Migaj, Marcin Gruchała, Małgorzata Lelonek

**Affiliations:** 1 First Department of Cardiology, University Clinical Centre, Medical University of Gdansk, Smoluchowskiego 17 Str, 80-214 Gdansk, Poland; anna.kowalczys@gumed.edu.pl (A.K.); marcin.gruchala@gumed.edu.pl (M.G.); 2 Department of Coronary Disease and Heart Failure, St. John Paul II Hospital, 31-202 Kraków, Poland; j.nessler@szpitaljp2.krakow.pl; 3 Department of Coronary Disease and Heart Failure, Institute of Cardiology, Faculty of Medicine, Jagiellonian University Medical College, 31-202 Kraków, Poland; 4 Department of Cardiology, University Hospital Poznan, Poznan University of Medical Sciences, 60-535 Poznan, Poland; ewa.straburzynska-migaj@skpp.edu.pl; 5 Department of Noninvasive Cardiology, Medical University of Lodz, 90-549 Lodz, Poland; malgorzata.lelonek@umed.lodz.pl

**Keywords:** epidemiology, heart failure, hospitalizations, mortality

## Abstract

*Background and Objectives*: Cardiovascular diseases (CVDs) remain the leading cause of mortality in Poland, with heart failure (HF) presenting a significant public health issue. *Materials and Methods*: This study aimed to analyze trends in HF incidence, hospitalization rates, patient demographics, and mortality over two decades A comparative analysis was performed using data from two national reports: (1) the 2013 report “Heart Failure—Analysis of Economic and Social Costs, “ assessing HF patients from 2004 to 2012, and (2) the 2023 report “Heart Failure in Poland 2014–2021,” based on data from the Polish Ministry of Health, National Health Fund, and HTA Consulting. This study examined the prevalence of HF (ICD-10 codes: I50, J81), hospitalization rates, comorbidities, mortality trends, and access to rehabilitation. *Results*: Between 2014 and 2019, the number of HF patients grew by 34%, reaching 1.02 million in 2019. Only 9% of HF patients were younger than 60 years. Multimorbidity was common, with arterial hypertension, atherosclerotic cardiovascular disease, and arrhythmias, often preceding HF diagnosis. HF-related mortality increased, with 149,963 in 2021, compared to 16,606 in 2012. In 2019, hospitalizations related to HF increased by 41% compared to 2014. The economic burden of HF care increased by 117% between 2014 and 2020, with hospitalizations accounting for 94% of total costs, up from 65% in 2012. Access to cardiac rehabilitation remained limited. *Conclusions*: HF prevalence, hospitalization rates, and mortality have increased in Poland, alongside a rising burden of multimorbidity. These findings provide a foundation for future healthcare planning to reduce the impact of HF in Poland.

## 1. Introduction

Cardiovascular diseases (CVDs) remain the leading cause of death in Poland [1,2]. Heart failure (HF) poses a significant health and socio-economic challenge within the country. Approximately 1.2 million patients are estimated to have symptomatic HF, accounting for about 3.2% of the general population [3]. According to the current definition, HF is diagnosed based on symptoms and signs resulting from structural and/or functional abnormalities of the heart, along with elevated natriuretic peptide levels and/or evidence of congestion in the pulmonary and/or systemic circulation, confirmed through objective methods [4]. The prognosis for this clinical syndrome remains poor, with the risk of death markedly increased among HF patients. It is estimated that up to 40% of patients with HF die within five years of diagnosis [5]. Data from the Central Statistical Office and the Polish Ministry of Health indicate that HF was the leading cause of all cardiovascular deaths in Poland in 2021. Additionally, around 140,000 patients die annually due to HF [1,5]. To improve HF prognosis, new treatment strategies, both pharmacological and invasive, have been introduced, enhancing patient survival as demonstrated in clinical studies [6]. Despite significant advances in diagnostics and treatments, HF continues to exert a substantial burden on Poland’s healthcare system.

This review aimed to present data on the epidemiology of HF in Poland by comparing two time periods: 2004–2012 and 2014–2021. It should be emphasized that this is the first study to examine HF in Poland from a 20-year follow-up perspective.

## 2. Materials and Methods

This review compares data on the incidence of HF and hospital admissions for patients with HF from 2004 to 2012 and from 2014 to 2021. To achieve this, two documents were analyzed. The first, titled “Heart failure—analysis of economic and social costs,” is a research report prepared by experts from the Institute of Management at Lazarski University in Warsaw, completed in 2013. It analyzed Polish patients with HF from 2004 to 2012 [7]. The second, titled “Heart Failure in Poland 2014–2021,” published in 2023, is a report issued by the Heart Failure Association of the Polish Society of Cardiology, developed in collaboration with the Ministry of Health of the Republic of Poland, the National Health Fund, the Analysis Department, and HTA Consulting [8]. The data extracted from the National Health Fund in Poland included patients diagnosed with HF (International Classification of Diseases, Tenth Revision (ICD-10) codes: I50, I50.1, I50.9) and with acute HF, coded as pulmonary edema (J81) [8].

## 3. Results

### 3.1. General Data

According to data from the Polish National Health Fund, 288,809 patients with HF were treated in Poland in 2012. The majority, 88,147 (31%), were diagnosed with I50.0 (congestive HF), followed by 88,029 patients (30%) with I50.9 (left ventricular failure, unspecified), 80,041 patients (28%) with I50 (HF), and 32,592 patients with I50.1 (left ventricular HF). Most services for HF patients were delivered through hospital care, totaling 187,481 cases (65%), followed by outpatient specialist services, which accounted for 93,356 (32%). Rehabilitation services were provided to 7972 (3%) HF patients.

**Figure 1:**
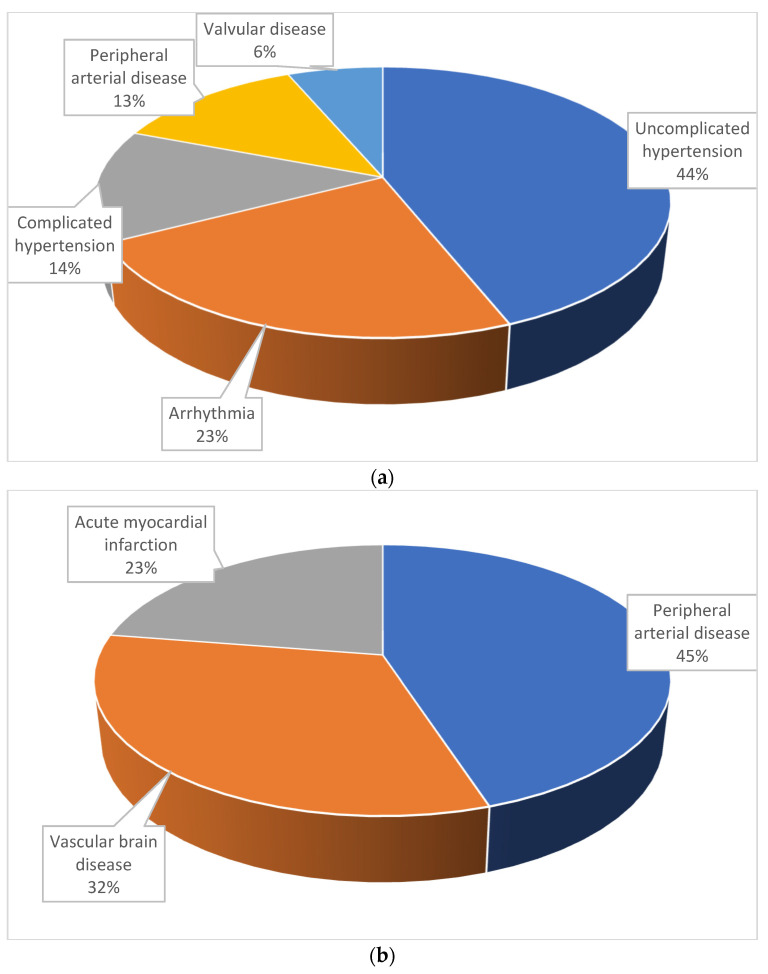
Most common diseases preceding HF in Poland 1 year before diagnosis—based on data from the Polish Ministry of Health from 2014 to 2021, according to the Elixhauser (**a**) and Charlson (**b**) multimorbidity index.

Based on the latest data published by the Ministry of Health, it has been shown that between 2014 and 2019, the number of patients diagnosed with HF (ICD-10: I50—congestive HF or J81—pulmonary edema) in Poland increased by 34% (from 167,000 to 223,000), reaching 1.02 million in 2019 [8]. There was a clear downward trend in the number of patients diagnosed with de novo HF, with 127,000 patients in 2019, representing a 26% decrease compared to 2014 [8]. According to data from the Ministry of Health, in 2021, the largest age group for HF prevalence remained among elderly patients, specifically those aged over 70 years (30%) and over 80 years (32%), with the average age of a patient with HF now being 75 years [8]. The phenomenon of multimorbidity is common in patients with HF [8]. Furthermore, it has been observed that multiple conditions often precede the onset of HF. According to data from the Ministry of Health from 2014 to 2021, the most common diseases preceding HF approximately one year before diagnosis in Poland mainly include other CVDs, such as arterial hypertension, atherosclerotic cardiovascular disease (ASCVD), arrhythmias, and cerebrovascular disease (Figure 1) [8]. Additionally, other conditions, such as chronic obstructive pulmonary disease (COPD), diabetes with and without complications, thyroid disease, and kidney disease, may be up to 15 per cent more common in patients with HF in the 12 months following the HF diagnosis, leading to their second hospitalization [8].

### 3.2. Mortality

**Figure 2:**
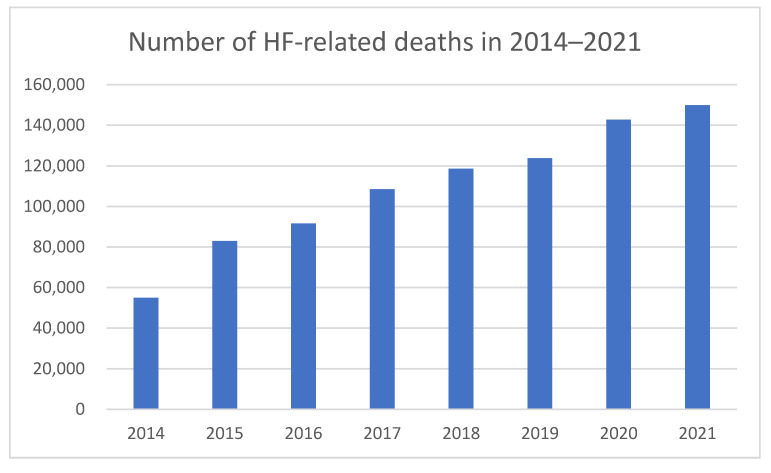
Number of HF-related deaths in 2014–2021.

**Figure 3:**
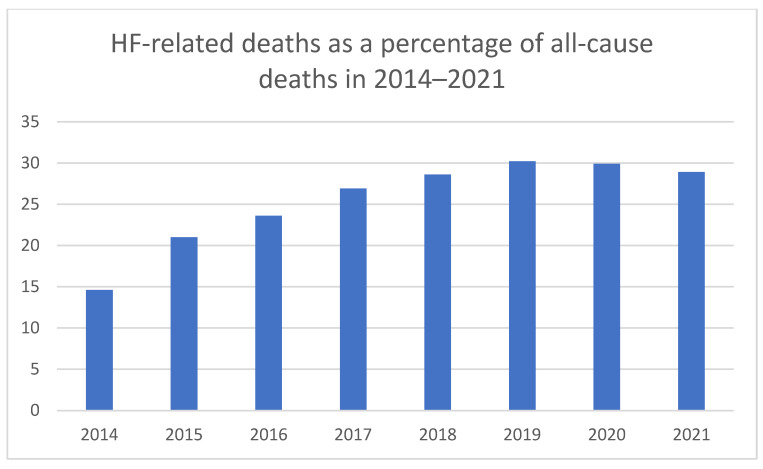
HF-related deaths as a percentage of all-cause deaths in 2014–2021.

Heart failure is the leading cause of cardiovascular deaths in Poland, with the number of fatalities caused by HF consistently increasing over the past 20 years. In 2021, there were 149,963 deaths attributed to HF, accounting for 28.9% of all deaths [8] (Figure 2 and Figure 3). In comparison, in 2014, the reported deaths caused by HF amounted to 55,003, representing 14.6% of all deaths [8] (Figure 2 and Figure 3). It is worth emphasizing that among patients in group E53 (HF > 69 years or with complications and comorbidities), the reported deaths amounted to 16,606 in 2012, with the mortality rate for hospitalized patients in this group at 11.44% [3].

Data from Poland covering 2014 to 2021 show that only 57% of patients survive for another five years after their HF diagnosis, regardless of the NYHA class. The median survival for patients aged 75 years and older is around four years from the time of diagnosis [8]. Moreover, Polish data reveal concerning trends in the prognosis of HF patients, with a declining chance of one-year survival from diagnosis, decreasing from 86% in 2014 to 76% in 2021 [8].

### 3.3. Hospitalizations

In 2012, Poland had an average of 376 hospitalizations per 100,000 people. Patients with HF were primarily admitted due to emergencies, which accounted for 82.97% of all hospitalizations. A report by the National Health Fund in Poland on hospital admissions for patients over 69 years old showed that in 2009, cardiac conditions made up 11% (232,723) of all hospital admissions among those aged 65 and older. In 2009, congestive HF (I50.0) was the most common cause of hospitalization in Poland, while unspecified HF (I50.9) was the second most common for both women and men over 69 years. The third most frequent diagnosis of HF, left ventricular HF (I50.1), was ranked as the tenth cause of hospitalization for women over 69 years and the thirteenth for men in this age group [3]. In 2012, among hospitalized patients in the E53 group, women (55.68%) slightly outnumbered men (44.32%) in terms of gender. Regarding age, most patients in this group were between 61 and 80 years old (51.27%), with those aged 81 years and above making up 43.69%.

According to the Ministry of Health, in 2019, the number of hospitalizations for HF, whether as a primary or secondary diagnosis, was 1022 per 100,000 population, while the number of hospitalizations with a primary diagnosis of HF was 726 per 100,000 population. A decrease in this indicator was observed between 2020 and 2021. The incidence of hospitalizations due to HF increased with age: from 19 per 100,000 in the 18–39 age group to 7448 per 100,000 in the 80+ age group. In the 60–69 age group, it exceeded 1000 per 100,000 population in 2018 and rose sharply in the subsequent age groups (70–79 and over 80). Table 1 presents the basic data on hospitalizations of HF patients in the compared age ranges.

**Figure 4:**
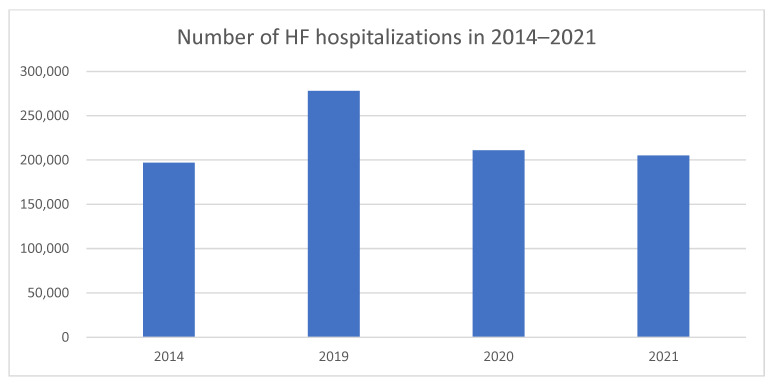
Comparison of HF hospitalization rates in 2014, 2019, 2020, and 2021.

It is important to note that the number of hospitalizations due to HF increased between 2014 and 2018, despite the decline in new cases. From 2014 to 2021, the number of hospitalizations with HF as the primary diagnosis ranged from 197,000 in 2014 to 278,000 in 2019, then decreased to 211,000 in 2020 and 205,000 in 2021 (Figure 4).

In each analyzed year, the number of hospitalizations due to HF exceeded the number of patients who received inpatient services (167,000–223,000) by 18–25%, indicating that the number of hospitalizations per patient requiring at least one hospitalization due to HF each year ranged from 1.18 to 1.25. Hospitalizations due to HF exacerbations serve as an important indicator of the severity of this clinical syndrome. Data from 2012–2018 show that the majority of hospitalizations due to HF (82%) were classified as emergency admissions. Between 2014 and 2019, the number of patients with HF (ICD-10: I50 or J81) experiencing at least one emergency hospitalization for the first time during HF increased, from 36,300 in 2014 to 44,000 in 2019. Additionally, the number of patients with HF who had at least two hospitalizations due to HF within 12 consecutive months also rose, with figures depending on the type of admission, from 33,200 to 45,100 in 2014, reaching 38,700 to 54,700 in 2019.

### 3.4. Rehabilitation and Palliative Care

Currently, only 0.55% of patients (i.e., approximately 1 in 180 patients) with HF in Poland participate in any form of rehabilitation within a calendar year through publicly funded services (before the COVID-19 pandemic, this rate was around 0.8% of patients) [8]. In the case of HF, rehabilitation was most often offered as part of inpatient services. Outpatient cardiac rehabilitation for patients with HF was very rarely provided [8].

Patients with HF did not receive palliative and hospice care services funded by the National Health Fund in Poland during the analyzed period.

### 3.5. HF Financial Burden

Between 2014 and 2019, expenditures related to HF as a principal diagnosis increased by approximately 117%. The primary cost was due to treating HF hospitalizations, which accounted for 94% of the total funds allocated for HF patient care. The remaining 6% was spent on nursing, care services, and outpatient specialized care [8]. Similarly, between 2004 and 2012, hospitalizations also accounted for 94% of all HF treatment costs [3]. The remaining percentage was used for rehabilitation services and outpatient specialized care [3].

## 4. Discussion

A comparison of data from reports on Polish patients with HF between 2004 and 2012 and between 2014 and 2021 shows the following conclusions: (1) the number of HF patients in Poland has increased significantly; (2) the number of hospitalized HF patients in Poland is rising; (3) it seems that the COVID-19 pandemic may have affected recent trends in HF patients.

A significant increase in the incidence of HF is expected in the coming decades due to an ageing population, an increase in the prevalence of HF risk factors such as atrial fibrillation and arterial hypertension, and more effective treatments for coronary artery disease [9]. Conversely, the prognosis for patients with HF is improving thanks to the growing use of new medications and innovative invasive procedures that reduce the risk of sudden cardiac death [10].

Data provided by the Ministry of Health indicated that between 2014 and 2019, the number of patients diagnosed with HF (I50 or J81) in Poland reached 1.02 million in 2019 [11]. During this period, a decrease in the number of patients with a de novo diagnosis of HF was observed, dropping to 127,000 in 2019, which represented a 26% decline from 2014 [11]. Previously published studies on HF reported an age-standardized prevalence of 1130 per 100,000 population. Compared to European Union countries, this is the fifth-highest rate [12,13]. It should be emphasized that, according to data from the Ministry of Health regarding the total number of patients in Poland, this indicator was significantly higher, reaching 2413 per 100,000 inhabitants in 2021, or 2626 per 100,000 inhabitants when adjusted for age, sex, and place of residence [11]. In 2021, individuals aged 80–89 years (32%) formed the most significant proportion of HF patients, followed by those aged 70–79 years (30%). Over 90% of patients were aged 60 years and older [13]. These findings align with other scientific reports that confirm the association between age and an increased incidence of HF [14,15,16].

According to reports, the one-year mortality rate after HF hospitalization in Poland remains high, with HF continuing to be one of the most significant contributing factors. Similarly, recently published data indicate that mortality rates in HF have been steadily rising over the past decade [17,18]. The COVID-19 pandemic and the underuse of guideline-directed medical therapy are suggested as potential reasons for the increasing mortality in this group [17,18,19]. Other studies have also found that 35.2% of patients aged 65 and older, who were hospitalized for HF, died within 12 months of discharge [20]. Furthermore, data from the analyzed reports show that only 57% of patients survive five years after their diagnosis of HF, regardless of the NYHA class. Recent reports by Shah KS et al. also highlight the poor prognosis in HF, with a five-year mortality rate of 75.3%, 75.7%, and 75.7% in HF with reduced ejection fraction (HFrEF), HF with mildly reduced ejection fraction (HFmrEF), and HF with preserved ejection fraction (HFpEF), respectively [21]. Rywik et al. observed that the survival rate at 1 and 5 years for HFrEF, HFmrEF, and HFpEF was 81%, 84%, and 84%, and 47%, 61%, and 59%, respectively [22]. In another study, 25.8% of HF patients (*n* = 6035) died during a median follow-up of two years, mainly due to cardiovascular causes [23].

Despite advances in treating HF, the rate of HF hospitalizations remains high, affecting both patients experiencing HF for the first time and those with exacerbations of chronic HF. An exacerbation of this clinical syndrome requiring hospital treatment is a significant adverse factor for prognosis, as it results from HF progression [14]. The number of hospitalizations for HF patients per 100,000 population serves as an important indicator that influences the organization of healthcare related to this condition. The hospitalization rate in a given area is affected by several factors, including the services provided in primary care, the availability of outpatient care and inpatient rehabilitation, the number of hospital beds—particularly in internal medicine and cardiology—and the terms of contracts with the National Health Fund. As discussed in the reports above, the frequency of hospitalization for HF increases with age (Table 1). Between 2014 and 2018, the number of hospitalizations rose despite a decrease in new cases. In the 60–69 age group, it exceeded 1000 per 100,000 population in 2018 and increased sharply in the subsequent age groups (70–79 and over 80 years old). According to data from 2012–2018, the majority of hospitalizations for HF (82%) occurred as emergency admissions. It can be inferred that this percentage has remained at a similar level since 2018. The available data do not permit an estimation of the rehospitalization trend.

Between 2020 and 2021, a decline in hospitalization rates was observed; however, this was not due to an improvement in the health status of HF patients but rather a result of limited healthcare access during the COVID-19 pandemic [24]. Significant changes in epidemiology took place during the first two years of the COVID-19 pandemic. The incidence and prevalence rates of HF decreased, along with a notable reduction in hospitalizations for HF and an increase in both overall and in-hospital mortality among HF patients, as highlighted by Zaleska-Kociecka et al. [19]. The annual decrease in the total number of hospitalizations exceeded 20%. The decline in hospitalizations in cardiac wards was considerably greater compared to non-cardiac wards [16].

This report examines the escalating direct costs related to heart failure (HF) management. The predominant expenditure was attributed to HF hospitalizations, accounting for 94% of the total funds allocated for HF patient care. Conversely, limited information is available concerning the indirect costs, such as productivity loss, associated with HF in Poland. Lyszczarz et al. demonstrated that the total indirect costs of HF in Poland amounted to EUR 871.9 million in 2012, increasing to EUR 945.3 million in 2015 [25]. Moreover, although HF is more frequently diagnosed in elderly populations, the productivity losses linked to HF within the working-age demographic in Poland represented 0.22% of the gross domestic product [25]. Recent analyses by Darvish et al. have also underscored the increasing costs associated with HF. The global economic burden of HF was estimated at USD 284.17 billion in 2021, comprising USD 136.86 billion (48.16%) in direct costs and USD 147.31 billion (51.84%) in indirect costs [26].

Although recognized as a key part of care for hemodynamically stable patients with heart failure (HF), cardiac rehabilitation (CR) was rarely available to Polish HF patients during the analyzed period. The lack of access to CR has been identified as a critical area for improvement in HF care in Poland. Other studies show that only 15% of CR referrals are made due to HF, and around 20% of HF patients prematurely end their CR programs [27]. Furthermore, research indicates that older age, female sex, and racial factors are linked with reduced access to cardiac rehabilitation in HF [28,29].

During the analyzed period, palliative and hospice care were seldom utilized in HF, as the National Health Fund did not reimburse these services. Currently, access to palliative care in Poland is steadily improving.

### 4.1. Future Recommendations

Given that heart failure (HF) poses a significant socio-economic and epidemiological burden in Poland, additional measures are required to prevent new cases of HF, enhance survival rates, and reduce hospitalization risks. As conditions such as diabetes, chronic kidney disease, and hypertension are prevalent among HF patients and often precede HF, it is imperative to implement strategies aimed at limiting the progression of these diseases. Beyond lifestyle modifications and the management of blood pressure and lipid levels, the early administration of SGLT-2 inhibitors in patients with type 2 diabetes who are at high cardiovascular risk is recommended to prevent the onset of HF. Additionally, SGLT-2 inhibitors are advised for patients with type 2 diabetes and chronic kidney disease to mitigate the risk of HF hospitalization and cardiovascular mortality [6]. Furthermore, the European Society of Cardiology recommends finerenone for patients with type 2 diabetes and chronic kidney disease to mitigate the risk of heart failure hospitalizations [6]. In patients exhibiting symptoms of heart failure with reduced ejection fraction, mildly reduced ejection fraction, and iron deficiency, intravenous iron supplementation with ferric carboxymaltose or ferric derisomaltose should be contemplated to decrease the likelihood of heart failure hospitalization [6]. Consequently, increased utilization of SGLT2 inhibitors, finerenone, and intravenous iron supplementation among appropriately selected patients may enhance symptom management and reduce the incidence of heart failure hospitalizations within the Polish population.

Contemporary models of care in HF are evolving from multidisciplinary approaches to specialized disease-management clinics and home visitation programs conducted by HF nurses, which have demonstrated a reduction in all-cause mortality in comparison with standard care [30]. During the analyzed period, there was a lack of structured ambulatory care involving the proactive engagement of HF nurses. The integration of HF nurses into patient management and educational initiatives in Poland is anticipated to substantially reduce the incidence of HF-related hospitalizations. In addition, CR should be more widely offered to HF patients in Poland as it has been shown to reduce HF hospitalizations, improve exercise capacity, and quality of life [30].

### 4.2. Limitations of the Study

The primary limitation of this study resides in the heterogeneity of data concerning heart failure (HF) patients across the two reports. The report, covering the years 2004–2012, primarily included provider data related to funding for HF procedures, which were predominantly regionalized and reported separately for each province. Conversely, the 2014–2021 report primarily focused on HF as a public health concern, encompassing direct costs and HF care programs. When comparing these reports, the unavailability of raw data precluded the compilation of figures beyond those presented in the cited studies. Moreover, the reports did not address issues related to HF pharmacotherapy. Given the recent registration of new medications for HF treatment, such a comparison would have yielded valuable insights. Additionally, due to the analysis based on ICD-10 classification, it was not feasible to evaluate different HF phenotypes. Lastly, it is possible that the underuse of specific ICD-10 coding, particularly among deceased patients during the period under review, may have influenced the actual number of reported HF-related deaths. Despite these limitations, the factual validity of the data remains intact.

## 5. Conclusions

According to epidemiological projections in Poland, there has been a steady increase in the number of patients with HF over the past 20 years, as well as in the frequency of hospitalizations due to the condition. Heart failure poses a significant challenge for modern healthcare because of the ageing population, the rising prevalence of risk factors contributing to its development, and the extended survival of patients within this cohort.

## Figures and Tables

**Table 1:** Number of hospitalizations due to heart failure (HF) in 2012 and 2018 within comparable age ranges. The average population of Poland in 2012 was 38,533,299 inhabitants, whereas in 2018, it was 38,411,148 inhabitants.

Year	2021		2018	
Age (Years)	Number of Hospitalizations	Share (%)	Age (Years)	Number of Hospitalizations per 100,000 Inhabitants
19–40	223	0.15	18–39	19
41–60	7100	4.89	40–49	92
61–80	74,431	51.27	50–59	342
≥81	63,428	43.69	60–69	1056
			70–79	2873
			≥81	7448

## Data Availability

Not applicable.

## Abbreviations

The following abbreviations are used in this manuscript:

CR	cardiac rehabilitation
CVD	Cardiovascular disease
HF	Heart failure
